# Interspecific and intraspecific variation in grasshopper (Orthoptera: Acrididea) molar form: implications for dietary ecology

**DOI:** 10.1098/rsos.240596

**Published:** 2024-10-16

**Authors:** Michael A. Berthaume, Matthew J. Morley

**Affiliations:** ^1^ Department of Engineering, King’s College London, London, UK; ^2^ Division of Mechanical Engineering and Design, London South Bank University, London, UK

**Keywords:** dental topographic analysis, grasshopper, sexual dimorphism

## Abstract

Like many mammals, grasshoppers (infraorder Acrididea) chew using molariform structures. Despite decades of research on mammals, little is known about grasshopper molar form and how it relates to grasshopper feeding biomechanics, diet, dietary ecology and evolution. Here, we develop a method for quantifying molar form and apply it to two species of distantly related grasshoppers with different diets (*Phymateus saxosus*, seven females; *Valanga nigricornis*, seven females, 11 males). We show that there are quantifiable differences in molar form, potentially related to diet. There are some differences in molar shape between left and right molars in both species and sexes, and significant differences in molar size, potentially due to scaling. Like in mammals, molar wear can cause large differences in molar shape. Species differences in molar shape did not match what was expected based on mammalian molar functional morphology. Dental topographic analysis is a promising new avenue for quantifying molar form in grasshoppers and a distinct advantage over traditional two-dimensional microscopy methods, and promises to reveal much about the biology, biomechanics and evolution of Acrididea.

## 1. Introduction

Like many mammals, grasshoppers chew their food using primarily the molariform portion of their mandibles (a multi-cusped/lophed portion of the mandible morphologically resembling a mammalian molar, herein, molars). In 1944, Isely made a stunning observation: grasshoppers that eat primarily grass (graminivores) have bladed, mammoth-like molars, while grasshoppers that eat primarily leafy forbs (forbivores) have cusped, mastodon-like molars [1]. As there is no chance this is due to shared ancestry (endoskeleton versus exoskeleton, more than 900 million years divergence [2–4]), this was an exquisite example of convergent evolution. As mammoths were predominantly grazers, and mastodons were predominantly browsers, consuming leaves similar in structure to forbs, dental convergence was probably due to similarities in diet related to food item breakdown. Interestingly, this potential convergence has not been investigated since.

**Figure 1.**
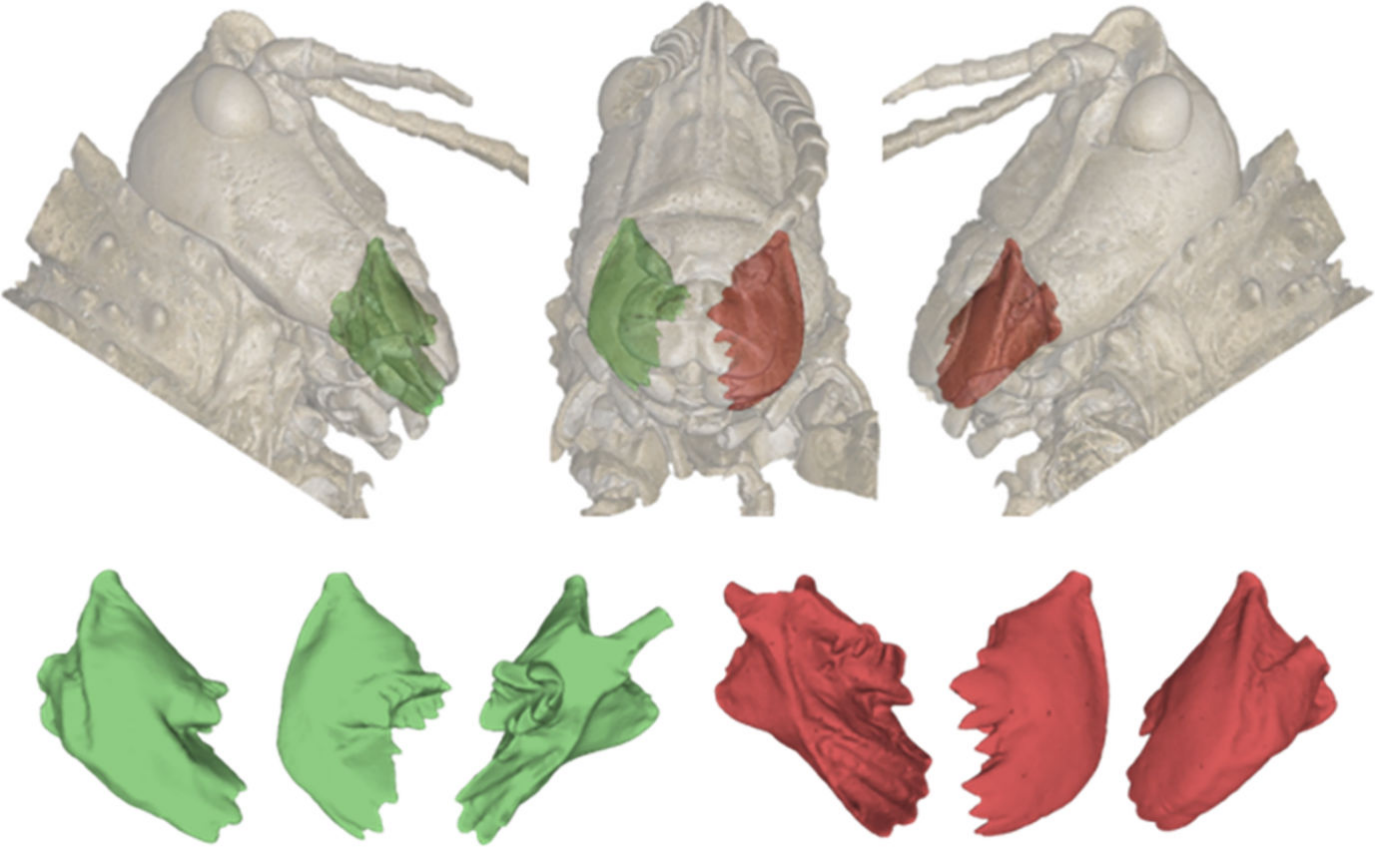
*Phymateus saxosus* head, highlighting the right (green) and left (red) mandibles. Mandibles have a ‘molar’ and ‘incisor’ region, along with a median molar [5] between the molar and incisor.

The shape of grasshopper mandibles and molars (figure 1) are often used in dietary studies, where mandibles are generally quantified using linear dimensions, and molars are described qualitatively [6–8]. Molar quantification is hindered by difficulties in (i) accurately and non-destructively capturing the three-dimensional geometry of low-density materials, such as chitin, at this scale and (ii) quantifying abstract shapes, such as molars, which can lack homologous landmarks [5]. Given the relationship between molar shape and diet, this severely limits our understanding of grasshopper feeding biomechanics, diet, dietary ecology and evolution [1,6–11], with applications in fields such as agriculture and conservation.

Here, we develop a method for quantifying grasshopper molar shape and apply it to grasshoppers to answer the following questions:

1. Do distantly related species with different diets have quantifiable differences in molar form (shape and size)?
2. Do interspecific differences in molar form converge with dietary-related differences in mammalian molar form?
3. What intraspecific variation exists in grasshopper molar form?

Previous research on grasshoppers has focused on the mandibles or qualitative descriptions of the molars. To our knowledge, this is the first study to quantify grasshopper molar shape. Based on previous studies [1,6] and work quantifying the gnathal edge of the mandible [12], we hypothesize molar form will differ between species. Given size sexual dimorphism in Orthoptera, mandibular sexual dimorphism in some clades (e.g. the enlarged ‘tusk’ mandibles in male Anostostomatidae, king crickets [13]), directional asymmetry in mandibles and changes in mandibular morphology with wear [5], we hypothesize that there will be significant intraspecific variation in grasshopper molar form, like in mammals (e.g. [14,15]).

## 2. Material and methods

Here, we assume that the main source of morphological variation (i.e. presence or absence of cusps, crests and basins) is due to genetics and that consuming different foods would not cause significant differences in molar morphology (e.g. for cusps to become crests).

### 2.1. Sample

Two extant species of grasshoppers, *Phymateus saxosus* (family Pyrgomorphidae, *n* = 7 females) and *Valanga nigricornis* (family Acrididae, *n* = 11 males, *n* = 7 females) were investigated. Taxa were chosen for their large size, differences in diet and phylogenetic distance (divergence: 130–104 Ma [16]), reducing the chances of resemblance due to phylogenetic influence. Grasshoppers in their final instar were purchased from breeding colonies (World of Butterflies and Moths, https://wobam.co.uk/; Insect Collector’s Shop, https://collectionneurdinsectes.com/; Alanscollectibles, https://www.etsy.com/uk/shop/Alanscollectibles?ref=l2-about-shopname). Unfortunately, grasshopper age was not known at the time of purchase.

*Phymateus saxosus*, the rainbow milkweed locust from Madagascar, is forbivorous, consuming milkweed. Its diet gives the grasshopper an unpleasant odour disliked by dogs, hence, the local name ‘dog grasshopper’ [17,18]. *Valanga nigricornis*, the short-horned or Javanese grasshopper from southeast Asia and Australia (local Malay name belalang kayu, meaning wood locust), is a mixed feeder, consuming forbs (rubber leaves), leaves of trees (teak leaves) and grasses (rice plants, oil-palm leaves) [19]. We chose to compare a pair of species with partially overlapping diets instead of a pair of species with distinct diets (e.g. forbivore versus graminivore) to test the ability of our method of molar shape quantification (below) at identifying subtler differences in molar morphology. Based on observations from Isely and subsequent grasshopper functional morphology studies (e.g. [1,5,20]), we expect *P. saxosus* to have higher-crowned, more cusp-like molars consistent with browsing mammals and *V. nigricornis* to have lower-crowned, flatter molars, with more crest-like structures.

### 2.2. Surface digitization

Specimen mandibles were closed, and opposing molar occlusal surfaces were touching, making it difficult to isolate the sclerotized occlusal surfaces during scanning. To open the mandibles, specimens were exposed to 20% isopropanol solution either directly (soaking) or indirectly (sealed in a container with a soaked paper towel) to relax specimens and make the joints pliable. Mandibles were opened using plastic tweezers, and a small piece of paper towel or parafilm was placed between the mandibles while they air-dried to prevent them from closing.

Grasshoppers were micro-computed tomography (micro-CT) scanned at the Natural History Museum, London, using their Nikon Metrology HMX ST 225 scanner (electronic supplementary material, figure S1 and video S1; no filter, 2000 projections, two frames per projection, 95 kV, 74 μA, exposure time 354 μs, resolution 8–9.58 μm). Micro-CT scans were used in lieu of surface scanning [12] as it allows for the three-dimensional surfaces of the molars to be captured, allowing portions of the molar surface that are occluded during 2.5-dimensional scanning, e.g. due to curved cusps or other aspects of mandibular anatomy (e.g. sensilla [21]) to be imaged.

### 2.3. Molar form quantification: dental topographic analysis

To quantify molar shape, we used dental topographic analysis (DTA). Dental topographic analysis is a landmark-free, homology-free method of shape quantification, making it particularly useful for quantifying distantly related species with functionally homologous structures [22–24]. It has been useful for relating tooth shape to diet in mammals (e.g. [25–27]), particularly when comparing teeth with disparate occlusal features at various wear stages [27,28].

Four topographic metrics were used: Dirichlet normal energy (DNE), relief index (RFI), orientation patch count rotated (OPCR) and ambient occlusion (portion de ciel visible (PCV)) [25,27,29–31]. Dirichlet normal energy quantifies surface curvature, which is correlated to molar sharpness [31]. Convex and concave curvatures for DNE are reported in electronic supplementary material, table S1 [32]. RFI (equation (2.1)) is used to quantify relative molar height [25,29], which can be used as a metric for hypsodonty.


(2.1) $$RFI=ln\;\left(\sqrt{\frac{3D_{surface\;area}}{2D_{projected\;area}}}\right)\;.$$

OPCR quantifies surface complexity and is used to estimate the number of ‘tools’ (i.e. crests, cusps and bumps) on the molar’s occlusal surface [27]. Finally, PCV quantifies the portions of the molar exposed to or hidden from ambient light shining on the molar from the occlusal direction and is used to estimate morphological wear resistance [30]. Additionally, three-dimensional surface area (SA) and two-dimensional projected occlusal area (OA) were included, as size is often correlated with diet in mammals and grasshoppers [31,33].

### 2.4. Scan processing

Reconstructed *.raw files were uploaded to ImageJ and exported as *.tiff stacks. Preprocessing of scans followed protocols developed by Morley & Berthaume [34], summarized here. Molars and portions of the left and right mandibles around the molars were segmented in 3D Slicer (https://www.slicer.org/). A previous study performed DTA on the gnathal edge of the mandible, from the incisor to molar regions (pars incisor, pars molaris and surrounding structures) [12]. Here, we conducted DTA on only the molar surface (pars molaris) as, like many mammals, grasshoppers use their incisors for ingestive behaviours such as initiating food item fracture and molars for masticatory behaviours, such as chewing [9]. By isolating the molar surface, we only quantify the portion of the mandible responsible for food item breakdown.

**Figure 2.**
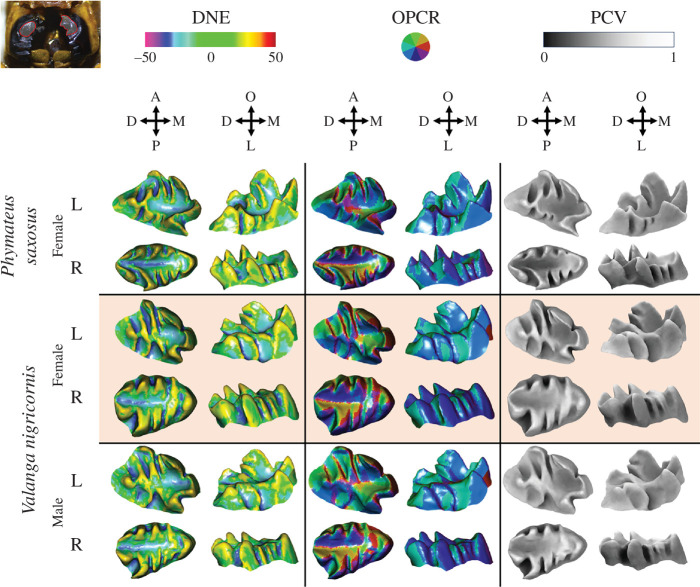
Occlusal and anterior (R molars)/posterior (L molars) plots of DNE, OPCR and PCV plotted on left and right *P. saxosus* and *V. nigricornis* molars (not to scale). Molars are remarkably similar in overall shape. Dirichlet normal energy on concave and convex surfaces are plotted as ‘negative’ and ‘positive’, respectively [32], but calculated using traditional DNE methods [31]. (Top left) Molar region (red outline) on black mandibles, with surrounding brown exoskeleton. Abbreviations: A, anterior; P, posterior; D, distal; M, mesial; O, occlusal; L, lateral.

Molar surfaces were imported into Meshmixer as *.stl files (https://meshmixer.com/) and cropped, isolating the entire outer molar surfaces (consistent with the entire enamel crown cropping method [22], which applied to taxa with enamel). Surfaces were imported into MeshLab as *.ply files and rotated to align the chewing (occlusal) surface with +*z*-axis (figure 2). Free-floating triangles were removed using the Remove Isolated Pieces (wrt Face Num) tool.

Many topographic metrics are summative and affected by triangle count [31,35,36]. Surfaces were decimated to approximately 10 000 triangles using MeshLab’s Quadric Edge Collapse Decimation tool (default settings except Quality Threshold 1, Preserve Normal selected). Surfaces were imported into R and RStudio as *.ply files using vcgPlyRead [37–39] and smoothed (vcgSmooth, Taubin smoothing, λ = 0.9, *μ* = −0.95, 10 iterations). The R package molaR [40] was used to remove floating vertices and faces with zero area (Molar_Clean) and quantify DNE, RFI and OPCR. For DNE, the top 1% energy × area values (DNE 99% [15]) were removed, and boundary triangles were included in the calculation [31]. Alpha = default and FindAlpha = TRUE were used for RFI [34], and two-dimensional projections were checked using the Check2D tool. Eight rotations of 5.625° and a minimum patch count of 3 were used for OPCR [27,41]. Surfaces were exported as *.ply files and imported into CloudCompare for PCV (count = 256, only the northern hemisphere selected). Plots of DTA results were checked individually for artefacts.

### 2.5. Intraobserver error

Isolating mammalian enamel crowns from micro-CT scans is simple, as enamel caps are significantly denser than the surrounding hard tissue. Grasshopper mandibular exoskeletons are continuous, and there is no definitive end to the molar region. As differences in segmentation and cropping can have significant effects on dental topographic measurements [25,31,42], we quantified interobserver error in segmentation and cropping using a single specimen. M.J.M. segmented and cropped the left molar of PS6 three times, waiting at least one month between attempts. Dental topographic and size metrics changed minimally (coefficients of variation: 0.003–0.041; see electronic supplementary material, table S2) demonstrating reliability in isolating molar surfaces for DTA.

Dirichlet normal energy and SA are insensitive to molar orientation, while others, such as RFI, OPCR, PCV and OA, are not [42]. Different methods for orienting mammalian teeth have developed, including maximum OA, using dentin horns and orienting teeth as if they were in the mandible [22,29,43].

The right and left molars of two female specimens (PS4 and VN11) were rotated clockwise and anticlockwise about the *x*- and *y*-axes just beyond the point where it could be reasonably said that the occlusal surface was aligned with the +*z*-axis, creating four new versions of each surface. Coefficients of variation for DNE (0–0.001), OPCR (0.035–0.092), RFI (0.057–0.115), PCV (0.005–0.014), three-dimensional SA (0) and two-dimensional projected area (0.049–0.106) were relatively low (electronic supplementary material, table S3).

### 2.6. Statistical analyses

Box and violin plots with a scatter overlay were used to visualize dental topographic values. Lines connected values for the left and right mandibles of the same individual. Relationships between topographic values were investigated using Pearson correlations. Paired Mann–Whitney *U*-tests with exact *p*-values were used when possible to test for differences between sides (left versus right molar) using the entire sample, within species and between sexes for the *V. nigricornis* sample (as only female *P. saxosus* were considered here). Unpaired Mann–Whitney *U*-tests with exact *p*-values (when possible) were used to test for differences in sex in left and right molar forms separately, using the *V. nigricornis* sample. The effect size was measured using Cohen’s *d* to quantify differences in values between our comparison groups.

We investigated more than seven individuals per group, as a minimum sample size of six is required to achieve an exact *p*-value less than 0.05. Coefficients of variation (population standard deviation divided by population mean) were calculated and compared for our larger (*n* = 11) and smaller (*n* = 7) samples to determine if increasing our samples would be likely to change results, but we found nearly identical coefficients of variation between groups. Cohen’s *d*, which is independent of sample size, further demonstrates (§3) that effects sizes were generally large (0.8 or higher in 20/23 cases) when significant differences (*p* < 0.05) were found and small (0.2 or lower in 14/19 cases) when significant differences (*p* > 0.05) were not found. The congruence between statistical significance and effect size provides further confidence that our samples were large enough to detect differences between samples.

Mixed effects generalized linear models (glm function) were used to investigate the effects of side, sex, species and individual on topographic variables and determine if there were species-level differences in topographic variables. Side, sex and species were treated as fixed effects and individual as a random effect to account for multiple measurements per individual equation (2.1).


(2.2) Topographicvariable=side+sex+species+(1|individual).

Eight models were run per topographic variable, consisting of all permutations of fixed effects, including none to model random chance. Models were compared using Akaike information criterion modified for small samples (AICc, model.sel function in the MuMIn package in R [44]) to determine which fixed effects affected which topographic variables. Statistics were run in R and RStudio [37,38] and visualized with the help of ggplot2 [45].

## 3. Results

**Figure 3.**
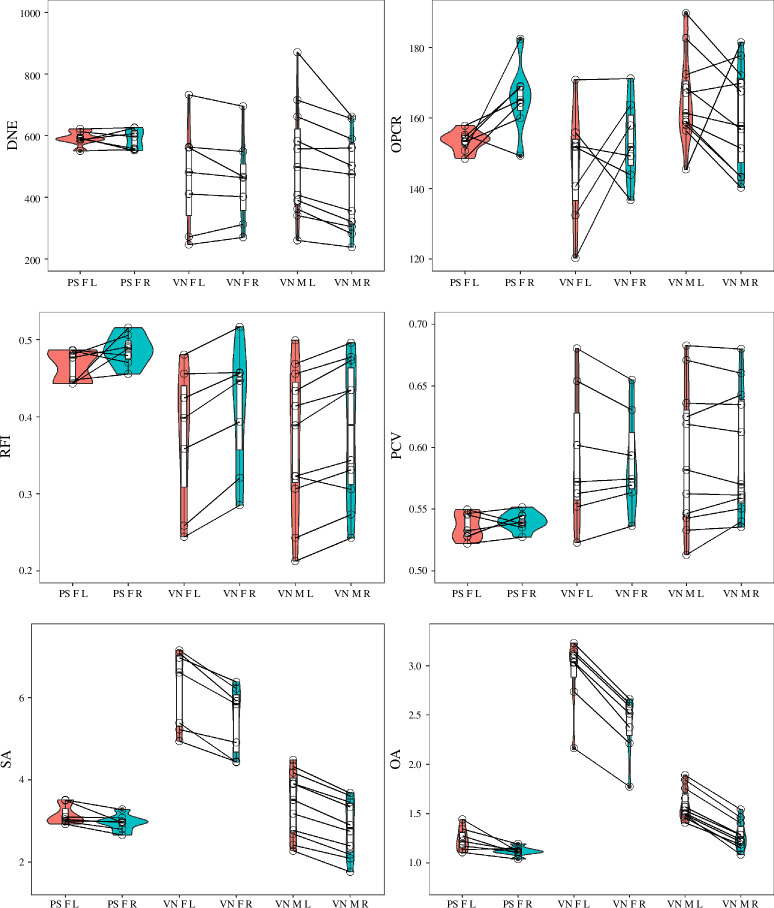
Violin and box plots. Abbreviations: PS, *Phymateus saxosus*; VN, *Valanga nigricornis*; F, female; M, male; L, left molar; R, right molar. Values for left and right molars for the same individual are connected.

There were quantifiable differences in molar shape between the forbivorous *P. saxosus* and mixed feeder *V. nigricornis*. On average, *V. nigricornis* molars had lower DNE, RFI and OPCR but higher PCV and were larger. Pairwise comparisons showed significant differences in size between sides (Cohen’s *d* > 0.8; table 1; figure 3), with left molars being larger than right molars for both species. Often, left and right molars differed in DNE and RFI but had similar OPCR and PCV. Within *V. nigricornis*, females had larger molars, and other than complexity (quantified using OPCR) of the left molars, there were no differences in molar shape between males and females. When differences between sides and sexual dimorphism existed, the effect was medium (Cohen’s *d* = 0.5–0.8) or large (Cohen’s *d* > 0.8; table 1; figure 3).

**Table 1.** Mann–Whitney *U*-tests.

					DNE		RFI		OPCR	
independent variable	species	sample	sex	side	*U* (*p*)	Cohen’s *d*	*U* (*p*)	Cohen’s *d*	*U* (*p*)	Cohen’s *d*
side	both	25	both	—	269 (0.003)	0.592	13 (0)	0.868	143 (0.615)	0.101
side	*P. saxosus*	7	both	—	14 (1)	0	5 (0.156)	0.536	1 (0.031)	0.814
side	*V. nigricornis*	18	both	—	154 (0.002)	0.745	2 (0)	0.949	97 (0.64)	0.11
side	*V. nigricornis*	11	male	—	65 (0.002)	0.934	1 (0.008)	0.841	51 (0.123)	0.465
side	*V. nigricornis*	7	female	—	18 (0.578)	0.21	0 (0.016)	0.914	9 (0.469)	0.274
sex	*V. nigricornis*	18	—	left	35 (0.791)	0.062	41 (0.86)	0.042	11 (0.014)	0.577
sex	*V. nigricornis*	18	—	right	37 (0.93)	0.021	41 (0.601)	0.123	30 (0.479)	0.167

					PCV		three-dimensional surface area (SA)		two-dimensional occlusal area (OA)	
independent variable	species	sample	sex	side	*U* (*p*)	Cohen’s *d*	*U* (*p*)	Cohen’s *d*	*U* (*p*)	Cohen’s *d*
side	both	24	both	—	134 (0.458)	0.148	300 (0)	1.059	299 (0)	1.033
side	*P. saxosus*	7	both	—	9 (0.469)	0.274	28 (0.016)	0.914	27 (0.031)	0.814
side	*V. nigricornis*	17	both	—	78 (0.766)	0.07	153 (0)	1.019	153 (0)	1.019
side	*V. nigricornis*	10	male	—	27 (0.638)	0.142	55 (0.002)	0.934	55 (0.002)	0.934
side	*V. nigricornis*	7	female	—	16 (0.813)	0.09	28 (0.016)	0.914	28 (0.016)	0.914
sex	*V. nigricornis*	18	—	left	40 (0.93)	0.021	77 (0)	0.943	77 (0)	0.943
sex	*V. nigricornis*	17	—	right	39 (1)	0	70 (0)	0.915	70 (0)	0.915

**Figure 4.**
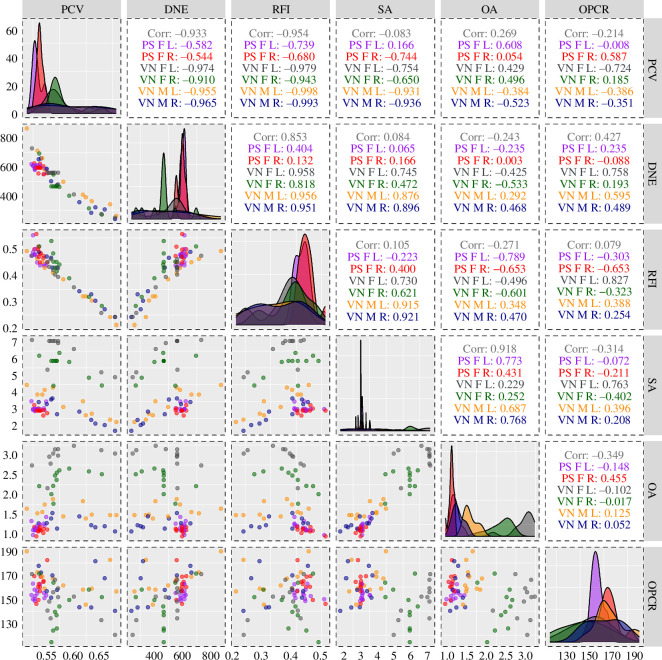
Pearson pairwise correlations between topographic variables. Corr: was calculated using the entire dataset. Data are the same as given in figure 3 legend.

Compared with *V. nigricornis*, there was relatively little variation in *P. saxosus* (table 2; figures 2 and 3; electronic supplementary material, table S1), potentially because *P. saxosus* molars were all relatively unworn, but *V. nigricornis* molars had variable levels of wear (electronic supplementary material, table S1; electronic supplementary material, figure S2). As in mammals, more worn molars had lower, rounded and flatter cusps and crests; consequently lower DNE, RFI, OPCR and three-dimensional SA; and higher PCV [30]. Pearson correlations revealed high levels of correlation between topographic variables, but the magnitude (e.g. DNE and three-dimensional SA) and direction (e.g. OPCR and three-dimensional OA) of the correlation sometimes changed, depending on if the entire dataset or a subset of data were being analysed (figure 4).

**Table 2.** Descriptive statistics for topographic variables. s.d. = standard deviation.

species	sample^a^	sex	side	DNE	OPCR	RFI	PCV	three-dimensional SA	three-dimensional OA
				mean (**s.d.**)	mean (s.d.)	mean (s.d.)	mean **(s.d.)**	mean **(s.d.)**	mean **(s.d.)**
*P. saxosus*	7	F	L	591.518 (22.8)	153.411 (3.065)	0.466 (0.021)	0.536 (0.011)	3.153 (0.247)	1.243 (0.12)
*P. saxosus*	7	F	R	588.674 (33.181)	165.589 (10.069)	0.487 (0.02)	0.54 (0.008)	2.963 (0.196)	1.118 (0.047)
*P. saxosus*	7	F	both	590.096 (27.391)	159.5 (9.542)	0.476 (0.022)	0.538 (0.009)	3.058 (0.236)	1.181 (0.109)
*V. nigricornis*	7	F	L	466.878 (172.272)	146.286 (16.637)	0.374 (0.093)	0.592 (0.057)	6.197 (0.97)	2.922 (0.369)
*V. nigricornis*	7	F	R	451.183 (143.839)	153.501 (11.787)	0.411 (0.083)	0.589 (0.041)	5.451 (0.835)	2.391 (0.313)
*V. nigricornis*	7	F	both	459.03 (152.685)	149.894 (14.349)	0.393 (0.087)	0.591 (0.048)	5.824 (0.952)	2.657 (0.429)
*V. nigricornis*	11	M	L	513.596 (184.578)	165.399 (12.608)	0.37 (0.095)	0.592 (0.058)	3.423 (0.798)	1.599 (0.164)
*V. nigricornis*	11	M	R	449.643 (155.416)	160.114 (14.572)	0.381 (0.093)	0.595 (0.052)	2.819 (0.69)	1.285 (0.144)
*V. nigricornis*	11	M	both	481.62 (169.695)	162.756 (13.569)	0.375 (0.091)	0.594 (0.054)	3.135 (0.792)	1.449 (0.22)
*V. nigricornis*	18	both	L	495.428 (176.251)	157.966 (16.826)	0.372 (0.091)	0.592 (0.056)	4.502 (1.626)	2.113 (0.71)
*V. nigricornis*	18	both	R	450.242 (146.667)	157.542 (13.599)	0.393 (0.087)	0.593 (0.047)	3.903 (1.521)	1.74 (0.603)
*V. nigricornis*	18	both	both	472.835 (161.436)	157.754 (15.08)	0.382 (0.089)	0.592 (0.051)	4.211 (1.582)	1.932 (0.677)

^a^ RFI could not be calculated for the right molar for one *V. nigricornis*, making the samples *n* = 10 for males and *n* = 17 for both.

Generalized linear models revealed that sex, side and species were always significant in predicting three-dimensional SA and two-dimensional OA and should be considered in studies of molar size (electronic supplementary material, table S4). No single model performed best for any shape metric (AICc > 4), but species was always included in the best-performing models for DNE, OPCR and PCV and often included in the best-performing models for RFI. Sex was always included in the best-performing models for OPCR, indicating there were sex and species differences in OPCR but potentially only species differences in the other shape metrics. The models with the highest weight were chosen for further consideration (table 3).

**Table 3.** Results from generalized linear models (GLMs). Positive values for side, sex and species indicate the right, males or *V. nigricornis* had greater values metric, respectively.

metric	intercept	side	sex	species
DNE	590.096	—	—	−117.261
RFI	0.476	—	—	−0.097
OPCR	159.5	—	12.863	−9.606
PCV	0.538	—	—	0.054
three-dimensional SA	3.321	−0.526	−2.702	2.766
two-dimensional OA	1.342	−0.322	−1.215	1.476

## 4. Discussion

For the first time, grasshopper molar form was quantified, and interspecific and intraspecific differences in molar form were quantified, and we were able to demonstrate the significance of both interspecific and intraspecific variation in determining molar form (shape and size; figure 2). This study demonstrates the usefulness of methods such as DTA in investigating mastication in non-tetrapods and in chewing invertebrates, such as grasshoppers, and addressing questions regarding the evolution, biomechanics and function of chewing molars.

On average, the more forbivorous *P. saxosus* had sharper, more complex and smaller molars that were more wear resistant and taller crowned than the mixed-feeding *V. nigricornis* (figure 3). This was unexpected as leaf-eating mammals, such as browsers, tend to have less complex molars than grazing mammals (e.g. [46]). Wear resistance is hypothesized to be more important to grazers, which eat more grass and potentially wear their teeth more by ingesting of silicon phytoliths and grit (e.g. [46–48]). There were some differences in molar shape due to side and sexual dimorphism, but differences were inconsistent and depended on the sample (table 1). Sexual dimorphism and sidedness in molar size could be due to scaling. Generally, female grasshoppers are larger and molar size scales with body size in animals, such as mammals [49]. Left mandibles overlap the right ones, making the left mandibles slightly larger [1].

Differences in molar size could also be related to diet, as graminivorous grasshoppers tend to have relatively larger heads than non-graminivores [33,50,51] due to relatively larger mandibular muscles [51] and mandibles [52]. This appears to be a functional adaptation to increase force production when fracturing grasses [50,53]. Bernays argued that the relatively larger mandibles and increased muscle mass are adaptations for mechanically challenging diets, as both C3 and C4 grasses are more mechanically challenging than herbaceous dicots [33,51,53]. However, the leaves from many woody plants are as mechanically challenging as C4 grasses [51], and Paine *et al.* demonstrated that some forb leaves can be more mechanically challenging than grasses [54].

We observed some differences in molar shape related to wear, but the lack of a method for quantifying molar wear in grasshoppers prohibited further investigation. Methods for quantifying mandibular wear exist, but these often use ratios or linear dimensions, which include measurements of incisal height (e.g. incisor height to hinge length). This makes these methods efficient at measuring overall mandibular, but not molar, wear [5,55,56]. It is possible that molar and incisal wear are perfectly correlated, but this has not yet been investigated. In mammals, molar wear is usually quantified using non-metric scales, which include dentin exposure (e.g. [57]), making them non-transferable here. It is possible that dental topographic metrics, such as RFI, which measure relative cusp height, could be used as a metric for dental wear, but the relief of an unworn molar would need to be known first as forbivores have relatively taller cusps, and graminivores have relatively shorter blades [1].

An interesting question that emerged from the results of this study is, ‘how important is molar wear, and how important is wear at different instar stages?’ In feeding experiments, Köhler *et al*. examined age-related wear in three species of short-horned grasshoppers (Acrididae) aged 0–7 weeks after reaching the final instar stage and found mandibular wear (measured as incisor length to mandibular width, meaning lower values correspond with more worn mandibles) decreased linearly with age in the individuals who were fed grass, but not Chinese cabbage or herbs, implying grass ingestion caused mandibular wear [58]. Chapman previously found a similar relationship between grass feeding and incisor : hinge ratio in locust [5]. Similarly, Kuřavová *et al*. found age-related differences in incisor length in two wild-caught species of Tetrigidae in their final instar stage, where age was estimated based on the date of specimen collection [55]. In leaf-cutter ants, mandible wear is correlated with cutting speed and energy expenditure [59], and extreme molar wear in mammals is correlated with decreased ability to sire young [60]. It is not unreasonable to hypothesize that molar wear is correlated with energy expenditure and potential evolutionary success in grasshoppers, where individuals with excessive molar wear prior to breeding will have lower evolutionary success than individuals without excessive molar wear. Indeed, wear-resistant molar adaptations, such as the inclusion of heavy metals in the cutting surface, have been shown to be important in the evolution of grasshoppers [61]. It is also possible that wear incurred in the molars of young grasshoppers will affect the shape of the molars and mandibles in later instar stages and, therefore, play an important role in determining the evolutionary success of a population or species. To our knowledge, this has not yet been investigated.

Despite more than 100 million years of separation [16], *P. saxosus* and *V. nigricornis* share remarkably similar dental morphologies. Both species have two large and one medium-sized ‘cusps’ on the mesial sides of the left mandibles, one of which is shaped similarly to a carnivoran carnassial. Large basins fill the centres of both molars. Going distally to mesially, right molars have four pairs of aligned cusps split by the basin, followed by the central cusp. Left molars have a central cusp followed by three pairs of aligned cusps split by the basin and another central cusp. In *V. nigricornis*, some of the pairs of cusps are connected across the basin to form the ridges (crests) commonly observed in graminivorous species, probably related to it being an intermediate feeder.

To our knowledge, the cusps and crests of grasshopper molars are not named, and no one has explicitly investigated their function. This, combined with the paucity of grasshopper-feeding kinematic studies, makes it difficult to interpret grasshopper molar functional morphology. It is similarly difficult to understand grasshopper molar evolution and whether similarities in dental form represent convergences, stabilizing selection or evolutionary constraints. Is evolution repeatedly selecting for molars with this bauplan, or is grasshopper molar growth and development highly genetically constrained?

This study represents a first step in better understanding grasshopper dental functional morphology. Stockey *et al*. applied DTA to the gnathal edge of 45 Orthopteran mandibles by removing the mandibles and taking 2.5-dimensional scans [12]. Their pioneering study highlighted the application of DTA in Orthoptera. Using three-dimensional micro-CT scans, we non-destructively captured portions of the chitinous gnathal edge that are hidden in 2.5-dimensional scans. Focusing on the molar surface, we constrain our analysis to portions of the mandible responsible for food item breakdown.

Here, we developed a method for quantifying grasshopper molar shape and used it to investigate interspecific and intraspecific variation in grasshopper molar form. By choosing taxa with similar molar form (despite being distantly related) and overlapping diets, we demonstrated the power of this method to quantify small but significant differences in morphology that are potentially related to diet. These analyses demonstrate the potential for DTA to quantify grasshopper molar shape as, despite sometimes large levels of intraspecific variation, the species-level variation comes through as the most important predictor of molar form (electronic supplementary material, table S4).

Limitations of this study include the use of insects from breeding colonies with unknown pedigree, meaning they could have been inbred, reducing intraspecific morphological variation. Without knowledge of the age of specimens or their diet, it is possible some of the differences in dental topographic values, particularly in the *V. nigricornis* sample, could be due to differences in age or diet, where individuals with more worn molars were older and/or raised on a more mechanically challenging diet. Certainly, age and diet affect mandible shape in grasshoppers and, therefore, probably affect molar shape [55,58]. As in mammals, the inclusion of variably worn molars, potentially a product of the sample being of mixed age and diet, appears to expand the range of dental topographic values for a species and/or reduce the chance of capturing interspecific differences. The use of mixed-age and mixed-diet individuals is common in mammalian dental topographic studies, where it is generally more important to use skeletal material from wild specimens. This reduces the risks of individuals being inbred with the added benefit that any changes in dental morphology due to wear are from individuals living on a natural diet. Finally, the use of dried specimens may cause shape changes in the molar, as the chitin may have warped due to loss of moisture.

## 5. Conclusions

Despite hundreds of millions of years separating them, grasshoppers and mammals have evolved functionally analogous mouthparts, including molars [20]. Here, we developed a method for quantifying grasshopper molar form, and by applying it to two distantly related species of grasshoppers, we demonstrate its robusticity and flexibility in quantifying differences in molar form. These differences do not appear to converge with dietary-related differences in mammalian molar form, but more data are needed. Intraspecific variation in molar shape can be high and is variably affected by sex, side (left versus right mandible) and molar wear. This makes DTA of grasshopper molars an exciting new avenue to investigate grasshopper feeding biomechanics, diet, dietary ecology and evolution.

## Data Availability

We have provided all data in the electronic supplementary material [62].
